# Flow cytometric lyophilised reagent tube assay for peripheral blood neutrophil myeloperoxidase expression to rule out myelodysplastic neoplasms at a university hospital: a diagnostic accuracy study

**DOI:** 10.1136/bmjopen-2024-095640

**Published:** 2025-08-22

**Authors:** Charlotte Planta, Laura Scheffen, Marie-Christine Jacob, Gautier Szymanski, Simon Chevalier, Sylvie Tondeur, Bénédicte Bulabois, Mathieu Meunier, Christine Lefebvre, Nicolas Gonnet, Frédéric Garban, Lysiane Molina, Claire Paradis, Arnaud Seigneurin, Radu Chiriac, Raymond Merle, José Labarère, Sophie Park, Tatiana Raskovalova

**Affiliations:** 1 Univ. Grenoble Alpes, CNRS, CHU Grenoble Alpes, Grenoble INP, TIMC, Grenoble, France; 2 Laboratoire d’immunologie, Centre Hospitalier Universitaire Grenoble Alpes, Grenoble, France; 3 Univ. Grenoble Alpes, CNRS, Inserm, CHU Grenoble Alpes, IAB, Grenoble, France; 4 Laboratoire d’hématologie biologique, Centre Hospitalier Universitaire Grenoble Alpes, Grenoble, France; 5 Service d’hématologie, Centre Hospitalier Universitaire Grenoble Alpes, Grenoble, France; 6 Univ. Grenoble Alpes, Inserm, CHU Grenoble Alpes, CIC, Grenoble, France; 7 Registre du Cancer de l’Isère, La Tronche, France; 8 Service d’hématologie biologique, Groupement Hospitalier Sud, Hospices Civils de Lyon, Lyon, France; 9 Univ. Grenoble Alpes, Département Universitaire des Patients, Grenoble, France; 10 Institute of Applied Healthcare Research, University of Birmingham, Birmingham, UK; 11 Clinical Epidemiology Unit, Centre Hospitalier Universitaire Grenoble Alpes, Grenoble, France

**Keywords:** Leukaemia, Aged, Cross-Sectional Studies

## Abstract

**ABSTRACT:**

**Objectives:**

Although flow cytometric analysis of peripheral blood neutrophil myeloperoxidase expression can accurately rule out myelodysplastic neoplasms (MDS), it lacks reliability and efficiency due to the practical limitations of laboratory-developed liquid reagent-based assays. This study aimed to quantify the agreement and comparative discriminatory accuracy between a single-use flow cytometric lyophilised reagent tube (BD Lyotube Stain 468) and its laboratory-developed liquid reagent counterpart.

**Design:**

Cross-sectional diagnostic accuracy study of two index tests against a reference diagnosis.

**Setting:**

A university hospital in France.

**Participants:**

Consecutive adult patients with an indication for bone marrow aspiration due to suspected MDS and unexplained peripheral blood cytopenia.

**Primary outcome:**

MDS confirmed by cytomorphological evaluation of the bone marrow aspirate performed in duplicate by experienced haematopathologists blinded to the index test.

**Results:**

Of 103 participants enrolled between July 2020 and August 2021, 37 had MDS (prevalence, 36%). The median intra-individual robust coefficient of variation (RCV) for myeloperoxidase expression was 30.9% using the BD Lyotube Stain 468 and 31.2% using the laboratory-developed liquid reagent assay, with an intraclass correlation coefficient of 0.94 (95% CI 0.91 to 0.96). The areas under the receiver operating characteristic curves were 0.83 (95% CI 0.74 to 0.90) and 0.82 (95% CI 0.73 to 0.89), respectively. Using a prespecified threshold of 30.0%, the corresponding sensitivity estimates were 89% (95% CI 75% to 97%) and 95% (95% CI 82% to 99%).

**Conclusion:**

BD Lyotube Stain 468 performs as well as its laboratory-developed liquid reagent counterpart for the quantification of myeloperoxidase expression by peripheral blood neutrophils. It may obviate the need for invasive bone marrow aspiration in up to 40% of patients with suspected MDS.

**Trials registration number:**

NCT04399018.

**Strengths and limitations of this study**

- The reference diagnosis was adjudicated in duplicate by experienced haematopathologists who were blinded to the index test results, with near-perfect inter-rater agreement.
- A prespecified threshold for both index tests was used to prevent reporting of optimistic diagnostic accuracy estimates.
- Unselected consecutive patients were enrolled to minimise the potential for spectrum bias.
- Bone marrow karyotype and molecular profiling were available for only 73% and 37% of the study participants, respectively.
- This study was conducted at a single university hospital laboratory and the results may not apply to other settings.

## Introduction

 Myelodysplastic neoplasms (MDS), previously designated as myelodysplastic syndromes, are clonally heterogeneous haematopoietic neoplasms that predominantly affect older patients.^1^ They are characterised by persistent cytopenia and morphological dysplasia in one or more cell lineages, with the potential for transformation to acute myeloid leukaemia.^1^

Suspected MDS represents the most common indication for bone marrow aspiration in older patients with cytopenia of unknown aetiology.^2 3^ However, a significant proportion of patients are unnecessarily exposed to the discomfort and potential harm of bone marrow aspiration,^4^,^6^ due to the relatively low prevalence of the disease.^7^

Attempts have been made to develop valid and reliable assays based on peripheral blood samples to rule out MDS, without the need for invasive bone marrow aspiration.^8 9^ We have previously reported on the accuracy of peripheral blood neutrophil myeloperoxidase expression quantified by flow cytometric analysis for the diagnosis of MDS.^10^ Cytoplasmic expression of myeloperoxidase is a marker of degranulation of mature granulocytes, a classic dysplastic feature of MDS.^11^ We found that an intra-individual robust coefficient of variation (RCV) for peripheral blood neutrophil myeloperoxidase expression of less than 30% excluded MDS, with 100% sensitivity and 100% negative predictive value, suggesting that it could obviate the need for bone marrow aspiration in up to 35% of patients referred for suspected disease.^10 12^

Although promising, this laboratory-developed assay has practical limitations for routine use due to the need for specific expertise, lack of standardisation and instability of liquid reagents.^13 14^ Lyophilisation, a method used to stabilise premixed multicolour reagent cocktails in flow cytometry tubes (Lyotube), may address the reliability and efficiency issues inherent to laboratory-developed assays.^13^ Indeed, lyophilised reagent cocktails offer a simplified way to handle complex multicolour flow cytometry assays, with performance comparable to reference liquid cocktails for various clinical applications.^14 15^

The MPO-MDS-Develop study was designed to assess the agreement and comparative discriminatory accuracy of a single-use customised lyophilised cocktail in a flow cytometry tube (namely, BD Lyotube Stain 468) and its laboratory-developed liquid reagent counterpart in quantifying peripheral blood neutrophil myeloperoxidase expression in patients referred for suspected MDS. We hypothesised that the two methods would demonstrate a high level of agreement and comparable discriminatory accuracy in ruling out MDS.

## Methods

### Study design

We conducted a cross-sectional diagnostic accuracy study of two index tests compared with a reference diagnosis in unselected patients referred for suspected MDS. The rationale and design of the study have been published elsewhere.^16^ This paper complies with the 2015 update of the Standards for the Reporting of Diagnostic Accuracy Studies guidelines.^17^

### Patient and public involvement

One of the authors (RM) was a member of the Patient Representatives Department at the Université Grenoble Alpes School of Medicine. He reviewed the study protocol and was involved in the interpretation and the dissemination plan of the study results. Patients or their representatives were not involved in any other aspect of the study.

### Participants

Consecutive adults referred for suspected MDS were screened for eligibility at a single university hospital in France. Participants were adults (aged 18 years or older) with an indication for bone marrow aspiration for suspected MDS and unexplained peripheral blood cytopenia defined by a haemoglobin concentration of less than 120 g/L for female patients and less than 130 g/L for male patients, a platelet count of less than 150×10^9^/L, or an absolute neutrophil count of less than 1.8×10^9^/L. Patients were excluded from the study if they had a documented history of MDS, were hospitalised in an intensive care unit, were homeless, were incarcerated, were not affiliated with a social security system or were unable to understand study information due to language limitations, dementia or altered mental status.

### Index tests

Independent operators blinded to the reference diagnosis performed flow cytometric analysis of peripheral blood samples, using either BD Lyotube Stain 468 (BD Biosciences, San Jose, California, USA) or laboratory-developed liquid reagent cocktail.

#### Flow cytometer

We used a three-laser, eight-colour, BD FACSCanto-II flow cytometer (BD Biosciences), which was maintained and quality controlled according to the manufacturer’s instructions. The study followed the Franceflow standard operating procedure to standardise instrument setup.^18^ Photomultiplier tubes were adjusted and checked daily using Rainbow Calibration Particles (BD Sphero, BD Biosciences). The fluorescence compensation matrix was established using BD CompBeads (BD Biosciences).

#### Blood sample collection

Peripheral blood samples were collected in BD Vacutainer 5 mL K2E (EDTA) anticoagulant plastic tubes (Ref 368861, BD Diagnostics, Le Pont de Claix Cedex, France). Samples were stored at room temperature and processed on the day of collection.

#### BD Lyotube Stain 468

A 50 µL aliquot of peripheral blood was stained with BD Lyotube Stain 468 for 15 min at room temperature in the dark according to the manufacturer’s instructions. BD Lyotube Stain 468 is a single-use, customised, five-colour lyophilised cocktail in a standard 12×75 mm polystyrene flow cytometry tube manufactured by BD Biosciences for the purpose of this study. It contained five fluorochrome-conjugated dried reagents, including CD15-PerCP-Cy5.5 (clone HI98), CD11b-APC (clone D12), CD16-APC-H7 (clone 3G8), CD14-V450 (clone MФP9) and CD45-V500 (clone HI30).

Peripheral blood neutrophil myeloperoxidase expression was quantified using BD Lyotube Stain 468, with satisfactory intra-assay (online supplemental table 1) and interassay (online supplemental table 2) precision.^19^,^21^ Unprocessed samples were stable for up to 72 hours at 4°C, but not at room temperature (online supplemental table 3).

#### Laboratory-developed assay

A 50 µL aliquot of peripheral blood was incubated with a panel of five fluorochrome-conjugated liquid reagents for 15 min at room temperature in the dark. The panel included 2.5 µL CD15-PerCP-Cy5.5 (clone HI98), 2.5 µL CD11b-APC (clone D12), 2.5 µL CD16-APC-H7 (clone 3G8), 2.5 µL CD14-V450 (clone MФP9) and 2.5 µL CD45-V500 (clone HI30).

#### Fixation and permeabilisation

The fixation and permeabilisation phases were performed using the BD IntraSure kit (BD Biosciences) in three steps, with incubation at room temperature in the dark, as previously described.^22^ During the permeabilisation phase, 10 µL of MPO-PE (clone 5B8) was added for intracellular marker staining. Antibodies, Lyotube Stain 468, BD FACS Lysing Solution (BD Biosciences) and the BD IntraSure kit were obtained from BD Biosciences.

#### Cell acquisition and analysis

A minimum of 10 000 neutrophils was acquired and analysed using BD FACSDiva software. We used the same gating strategy as described in detail elsewhere.^16 22^ Myeloperoxidase expression in the peripheral blood neutrophil population within a patient was expressed as the RCV, which was calculated as the robust SD divided by the median fluorescence intensity. The robust SD was calculated based on the deviation of individual data points from the median of the peripheral blood neutrophil population. The intra-individual RCV was expressed as a percentage and reflects the variability of myeloperoxidase expression in the peripheral blood neutrophil population within a patient.^10^

### Reference diagnosis

The reference diagnosis was adjudicated in duplicate by experienced haematopathologists blinded to the index test results. They based their diagnosis on cell morphology and the percentage of blasts in the bone marrow. Disagreements were adjudicated by a third haematopathologist. Inter-rater agreement was near perfect for the diagnosis of MDS (Fleiss kappa coefficient, 0.96, 95% CI 0.89 to 1.00) and for the percentage of bone marrow blasts (intraclass correlation coefficient, 0.92, 95% CI 0.89 to 0.94).

According to current guidelines,^23^ the criteria for MDS diagnosis were (1) the presence of ≥10% dysplastic cells in any haematopoietic lineage, (2) the exclusion of acute myeloid leukaemia (defined by ≥20% peripheral blood or bone marrow blasts) and (3) the exclusion of reactive aetiologies of cytopenia and dysplasia. Morphological assessment was supplemented by bone marrow flow cytometric score,^24^ karyotype and targeted next-generation sequencing panel, when relevant.^25 26^ Categorisation of MDS subtypes included those with defining genetic abnormalities and those defined morphologically.^23^ Cases of MDS were categorised using the original and revised International Prognostic Scoring System,^27 28^ respectively. The reference diagnosis for chronic myelomonocytic leukaemia (CMML) was based on published prerequisite and supporting criteria.^23^ CMML was classified into two subgroups, CMML-1 and CMML-2, based on the percentage of blasts and promonocytes, respectively.^23^

The differential diagnosis of MDS included clonal cytopenia of undetermined significance (CCUS) and idiopathic cytopenia of undetermined significance (ICUS). CCUS and ICUS were distinguished from MDS by the absence of dysplasia and increased blasts on peripheral blood and bone marrow examination.^2 29^ CCUS was defined as persistent unexplained cytopenia of at least 4 months’ duration, with the presence of somatic mutations of myeloid malignancy-associated genes detected in the blood or bone marrow with a variant allele fraction of at least 2% and no significant dysplasia.^2 29^ ICUS was defined as persistent unexplained cytopenia without somatic mutation or significant dysplasia.^2 29^ Repeated bone marrow aspirate within 6–12 months of enrolment was proposed to patients with ICUS or inconclusive or uninterpretable bone marrow examination at baseline.^16^

### Sample size

Assuming an area under the receiver operating characteristics (ROC) curve point estimate close to 0.90 for BD Lyotube Stain 468, we calculated that a sample size of 82 participants, including 18 MDS cases (22% MDS prevalence), would provide a precision of ±0.10 (95% CI ranging from 0.80 to 1.00). Anticipating a 20% rate of uninterpretable or inconclusive bone marrow aspirates, we recruited an additional 21 patients, giving an overall sample size of 103 participants.^16^

### Statistical analysis

Descriptive summary statistics were used to report categorical variables as numbers and percentages and continuous variables as medians along with 25th and 75th percentiles or range. Baseline patient characteristics and peripheral blood parameters were compared according to MDS status using the Pearson χ² test, replaced by Fisher’s exact test when appropriate, for categorical variables and the non-parametric Wilcoxon test for continuous variables.

Multivariable logistic regression was used to assess the independent associations of MDS with intra-individual RCV for peripheral blood neutrophil myeloperoxidase expression. OR estimates were adjusted for demographics, including age and sex, as well as haemoglobin concentration, absolute neutrophil count, platelet count and laboratory features that were associated with MDS in univariable analysis with p values less than 0.10 (ie, C reactive protein, alkaline phosphatase and gamma-glutamyltransferase concentrations). We assessed the log-linearity assumption for continuous independent variables by using fractional polynomial functions. Assuming that data were missing at random, we performed multivariate imputation of missing values using chain equations. For this purpose, 50 imputed datasets were created with a total run length of 50 000 iterations and imputations performed every 1000 iterations. Missing values for the index tests or indeterminate values for the reference standard were not imputed.

We assessed the agreement in continuous intra-individual RCV between BD Lyotube Stain 468 and the laboratory-developed liquid reagent-based assay graphically by examining a scatterplot of the differences versus the means of the two variables with the limit of agreement superimposed.^30^ We checked for the absence of bias by performing a regression analysis of the differences as a function of the means. The intraclass correlation coefficient was used to quantify the absolute agreement in continuous intra-individual RCV between BD Lyotube Stain 468 and the laboratory-developed liquid reagent-based assay performed on the same peripheral blood sample. In addition, we estimated the kappa coefficient to measure the agreement in binary intra-individual RCV with a prespecified threshold of 30.0%. This threshold was used because a previous study showed that an intra-individual RCV value of less than 30.0% accurately ruled out MDS, with both sensitivity and negative predictive value point estimates of 100%.^10^

We evaluated the comparative accuracy of the intra-individual continuous RCV obtained with BD Lyotube Stain 468 and the laboratory-developed liquid reagent-based assay by calculating and comparing the areas under the ROC curves. Sensitivity, specificity, positive predictive value, negative predictive value and likelihood ratio point estimates were also reported along with 95% CIs for binary intra-individual RCV with a prespecified threshold of 30.0%.

Two-sided p values less than 0.05 were considered statistically significant. Analyses were performed with Stata Special Edition V.16.0 (Stata Corporation, College Station, Texas, USA).

## Results

**Figure 1:**
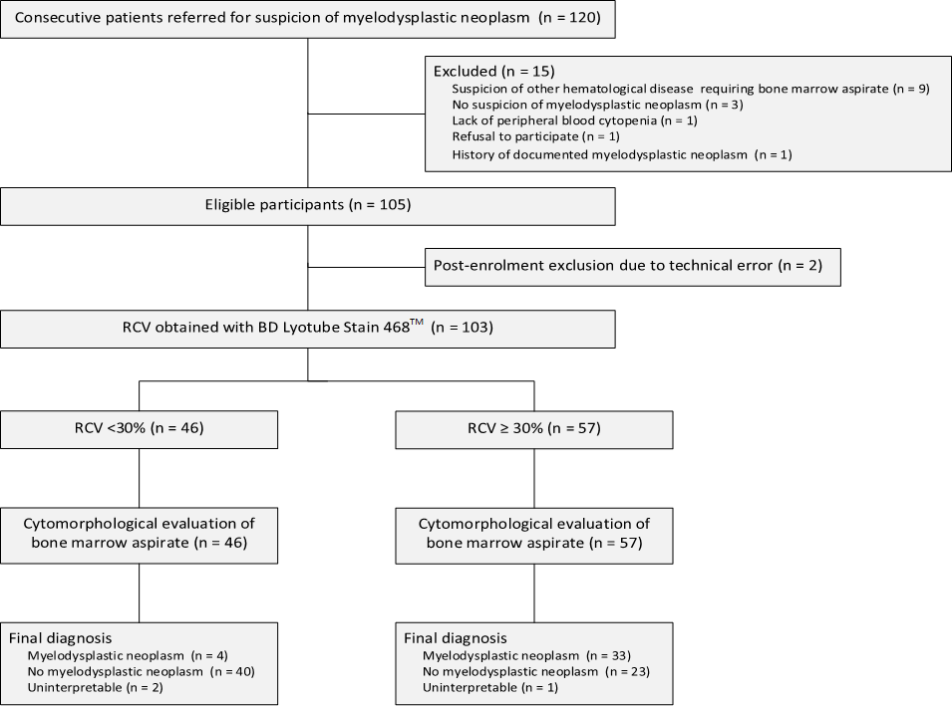
Flow of participants through the study. RCV, robust coefficient of variation for peripheral blood neutrophil myeloperoxidase expression.

Of 120 consecutive patients screened for eligibility between July 2020 and August 2021, 17 were excluded and 103 were successfully recruited into the study (figure 1). The median age of all participants was 76 years (25th–75th percentiles, 68–82) and 41 (40%) were female (table 1).

**Table 1:** Baseline clinical and laboratory features of participants (n=103)

Characteristics*		
Female gender, n (%)	41	(40)
Age, median (IQR), years	76	(68–82)
Smoking status, n (%)		
Never smoked	62	(60)
Former smoker	34	(33)
Current smoker	7	(6.8)
Alcohol use disorders, n (%)	14	(14)
Occupational exposure to chemicals, n (%)	3	(2.9)
History of haematological disease, n (%)†		
Lymphoproliferative syndrome	2	(1.9)
Multiple myeloma	1	(1.0)
Other	2	(1.9)
History of immunosuppressive or biological therapy, n (%)	8	(7.7)
Haemoglobin, median (IQR), g/L	105	(92–115)
Platelet, median (IQR), ×10^9^/L	136	(83–216)
White cell count, median (IQR), ×10^9^/L	4.7	(3.3–6.9)
ANC, median (IQR), ×10^9^/L	2.9	(1.9–4.4)
Lymphocytes, median (IQR), ×10^9^/L	1.2	(0.8–1.7)
Monocytes, median (IQR), ×10^9^/L	0.4	(0.3–0.6)
Reticulocytes, median (IQR), ×10^9^/L	72	(48–97)
Creatinine, median (IQR), µmol/L	77	(58–107)
Blood urea nitrogen, median (IQR), µmol/L	6.9	(5.1–11)
Bilirubin, median (IQR), µmol/L	11	(7–17)
AST, median (IQR), IU/L	25	(18–34)
ALT, median (IQR), IU/L	22	(15–29)
Gamma-glutamyltransferase, median (IQR), IU/L	44	(21–80)
Alkaline phosphatase, median (IQR), IU/L	76	(60–113)
Serum folic acid, median (IQR), ng/mL	9.1	(6.6–15)
Serum B12 vitamin, median (IQR), pg/mL	473	(361–733)
TSH, median (IQR), mIU/L	1.63	(1.06–2.35)
Haptoglobin, median (IQR), g/L	1.19	(0.64–1.76)
Ferritin, median (IQR), ng/mL	219	(97–505)
C reactive protein, median (IQR), mg/L	0	(0–15)

* Values were missing for blood urea nitrogen (n=2), bilirubin (n=2), alkaline phosphatase (n=1), gamma-glutamyltransferase (n=1), serum folic acid (n=2), TSH (n=1) and C reactive protein (n=1) concentration.

† No patient reported history of acute leukaemia, myeloproliferative neoplasms, idiopathic thrombocytopenic purpura, familial haematological malignancies, antineoplastic chemotherapy or radiation therapy. Other haematological diseases included minor thalassaemia (n=1) and haemochromatosis (n=1).

ALT, alanine aminotransferase; ANC, absolute neutrophil count; AST, aspartate aminotransferase; TSH, thyroid-stimulating hormone.

Cytomorphological evaluation of bone marrow aspirate confirmed suspected MDS in 35 patients at baseline. Two additional patients were diagnosed with MDS after repeat bone marrow aspiration for ICUS (one patient) or CCUS (one patient) during follow-up (online supplemental table 4). Overall, the analytical sample included 37 cases of MDS (prevalence, 36%), 63 unconfirmed suspicions of MDS (61%, including five patients with CCUS (4.9%) and three patients (2.9%) with ICUS) and 3 patients with uninterpretable bone marrow aspirate (2.9%). Bone marrow aspiration was repeated during the follow-up period and did not change the baseline reference diagnosis in three of five patients with CCUS, one of three patients with ICUS (online supplemental table 5) and one of three patients with an uninterpretable aspirate (online supplemental table 6). In addition, cytomorphological evaluation of the baseline or follow-up bone marrow aspirate helped to establish an alternative diagnosis in 4 of 63 patients (6.3%) with an unconfirmed suspicion of MDS (online supplemental table 7).

The median percentage of bone marrow blasts was 2.5%. MDS with low blasts accounted for 46% (17/37) of all MDS cases (table 2). Cytogenetic abnormalities were found in 53% (19/36) of MDS cases who underwent bone marrow karyotype analysis. Somatic mutations were detected in 96% (25/26) of MDS cases that underwent genomic sequencing analysis (online supplemental table 4). Overall, 10 MDS cases (27%) were classified as high or very high risk according to the revised International Prognostic Scoring System (table 2).

**Table 2:** Bone marrow features and 2022 WHO classification for confirmed suspicions of myelodysplastic neoplasms (n=37)

MDS features		
Bone marrow blasts, median (IQR), %	2.5	(1.8–5.5)
Any bone marrow cytogenetic abnormality, n (%)*	19/36	(53)
Any bone marrow somatic mutation, n (%)†	25/26	(96)
Bone marrow flow cytometric score≥2, n (%)‡	26/35	(74)
2022 WHO classification, n (%)		
MDS with defining genetic abnormalities		
MDS with low blasts and SF3B1 mutation (MDS-SF3B1)	4	(11)
MDS with low blasts and ring sideroblasts (wild-type SF3B1)	1	(2.7)
MDS with biallelic TP53 inactivation (MDS-biTP53)	1	(2.7)
MDS morphologically defined		
MDS with low blasts (MDS-LB)	17	(46)
MDS with increased blasts 1 (MDS-IB1)	5	(14)
MDS with increased blasts 2 (MDS-IB2)	5	(14)
Chronic myelomonocytic leukaemia (CMML1)	4	(11)
International Prognostic Scoring System, n (%)		
Low/intermediate-1	29	(78)
Intermediate-2/high	8	(22)
Revised International Prognostic Scoring System, n (%)		
Very low/low/intermediate	27	(73)
High/very high	10	(27)

* Bone marrow karyotype was not available for one patient.

† Molecular profiling was not available for 11 patients.

‡ Bone marrow flow cytometric^24^ score was computed as the number of parameters with values outside of the reference ranges among myeloblast-related cluster size in all nucleated cells (%), B-progenitor-related cluster size in all CD34+ cells (%), lymphocytes to myeloblast CD45 ratio and granulocyte to lymphocyte SSC ratio. Bone marrow flow cytometric score was not available for two patients.

MDS, myelodysplastic neoplasm.

Patients with confirmed suspicions of MDS were more likely to report a history of immunosuppressive or biological therapy. They had a lower median absolute neutrophil count and a lower median alkaline phosphatase concentration, but a higher median serum vitamin B12 concentration (online supplemental table 8). Other baseline clinical and laboratory characteristics showed comparable distributions for patients with confirmed and unconfirmed suspicions of MDS.

RCV values for peripheral blood neutrophil myeloperoxidase expression were determined for all 103 patients, with no missing values. The median time from baseline bone marrow aspiration to the performance of the index tests was 0 days (range, 0–1 day). The median intra-individual RCV values for peripheral blood neutrophil myeloperoxidase expression were 30.9% (25th–75th percentiles, 27.7–34.9) for BD Lyotube Stain 468 and 31.2% (25th–75th percentiles, 28.8–34.5) for the laboratory-developed liquid reagent-based assay. The mean difference in intra-individual RCV between the laboratory-developed assay and BD Lyotube Stain 468 was 0.6 percentage points, with 95% limits of agreement ranging from −4.4 to 5.7 (online supplemental figure 1).

The intraclass correlation coefficient estimate was 0.94 (95% CI 0.91 to 0.96), indicating a high level of agreement between BD Lyotube Stain 468 and its laboratory-developed liquid reagent counterpart for continuous intra-individual RCV. Agreement was also substantial for binary intra-individual RCV dichotomised using a prespecified threshold of 30%, with a percentage of agreement reaching 86% and an estimated kappa coefficient of 0.72 (95% CI 0.59 to 0.86, online supplemental table 9).

The analytical sample for the diagnostic accuracy of RCV consisted of 37 confirmed and 63 unconfirmed suspicions of MDS, after excluding three patients with uninterpretable bone marrow cytomorphology (figure 1). The median intra-individual RCV values for patients with confirmed and unconfirmed suspicion of MDS were 34.9% (range, 25.0–69.4) and 29.0% (range, 23.8–44.6) for BD Lyotube Stain 468 compared with 34.5% (range, 25.9–60.3) and 29.8% (range, 23.9–48.5) for the laboratory-developed liquid reagent-based assay, respectively (table 3). Both methods showed a significant trend towards higher RCV values for higher-risk MDS (online supplemental figure 2). The crude and adjusted ORs for confirmed suspicion of MDS associated with a 1% increase in intra-individual RCV were 1.29 (95% CI 1.15 to 1.46) and 1.39 (95% CI 1.17 to 1.66) for BD Lyotube Stain 468 and 1.24 (95% CI 1.11 to 1.38) and 1.34 (95% CI 1.14 to 1.57) for the laboratory-developed liquid reagent-based assay, respectively. Given that the prevalence of missing values was less than 2% for covariates (table 1), the OR estimates were consistent with and without multiple imputation of missing values.

**Table 3:** Comparative diagnostic accuracy of intra-individual robust coefficient of variation for peripheral blood neutrophil myeloperoxidase expression between a single-use flow cytometric lyophilised reagent tube (BD Lyotube Stain 468) and its laboratory-developed liquid reagent counterpart (n=100)*

	BD Lyotube Stain 468		Laboratory-developed liquid reagent test	
Intra-individual RCV, median (range), %				
Confirmed suspicion of MDS (n=37)	34.9	(25.0–69.4)	34.5	(25.9–60.3)
Unconfirmed suspicion of MDS (n=63)	29.0	(23.8–44.6)	29.8	(23.9–48.5)
RCV≥30%				
True positive, n	33	(…)	35	(…)
False positive, n	23	(…)	29	(…)
False negative, n	4	(…)	2	(…)
True negative, n	40	(…)	34	(…)
Sensitivity, % (95% CI)	89	(75 to 97)	95	(82 to 99)
Specificity, % (95% CI)	63	(50 to 75)	54	(41 to 67)
PPV, % (95% CI)	59	(45 to 72)	55	(42 to 67)
NPV, % (95% CI)	91	(78 to 97)	94	(81 to 99)
LR+ (95% CI)	2.44	(1.73 to 3.45)	2.05	(1.56 to 2.71)
LR− (95% CI)	0.17	(0.07 to 0.44)	0.10	(0.03 to 0.39)

* The analytical sample consisted of 37 confirmed and 63 unconfirmed suspicions of myelodysplastic neoplasm, after excluding three patients with uninterpretable bone marrow cytomorphology.

LR+, likelihood ratio of a positive result; LR−, likelihood ratio of a negative result; MDS, myelodysplastic neoplasm; NPV, negative predictive value; PPV, positive predictive value; RCV, robust coefficient of variation.

**Figure 2:**
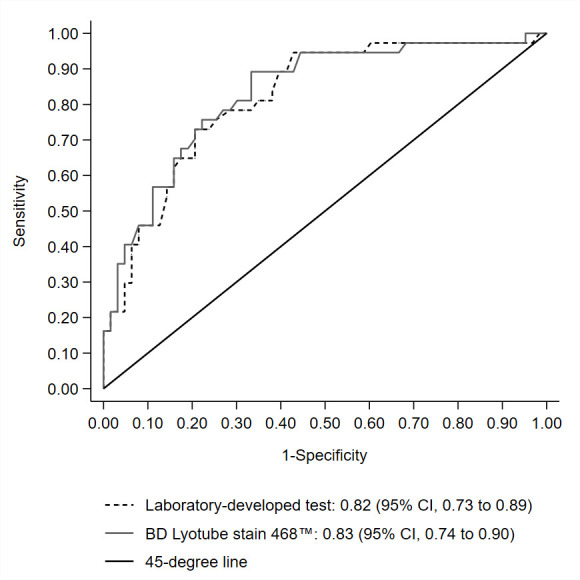
Comparison of area under the receiver operating characteristic curve of intra-individual robust coefficient of variation for peripheral blood neutrophil myeloperoxidase expression between a single-use flow cytometric lyophilised reagent tube (BD Lyotube Stain 468) and its laboratory-developed liquid reagent counterpart (n=100)^*^.^ *^The analytical sample consisted of 37 confirmed and 63 unconfirmed suspicions of myelodysplastic neoplasm, after excluding three patients with uninterpretable bone marrow cytomorphology.

Accordingly, the discriminatory accuracy of the continuous intra-individual RCV was comparable for BD Lyotube Stain 468 and its laboratory-developed liquid reagent counterpart. The area under the ROC curve point estimates was 0.83 and 0.82 (p=0.59; figure 2). Using a prespecified 30% threshold for RCV, there were two false negative MDS cases for the laboratory-developed liquid reagent assay and four for BD Lyotube Stain 468 (table 3). The corresponding sensitivity point estimates were 95% and 89% (p=0.50).

False negative cases had a median percentage of bone marrow blasts of 2.0 (range, 1.7–7.0). They were classified as very low (n=1), low (n=2), and high (n=1) risk according to the revised IPSS. Their median time from peripheral blood collection to flow cytometric analysis was 7h24 (range, 3h02–10h15) (online supplemental table 4). There were no adverse events associated with the performance of the reference standard or index tests.

## Discussion

This study demonstrated a substantial to high level of agreement between BD Lyotube Stain 468 and its laboratory-developed liquid reagent counterpart for binary and continuous RCV of myeloperoxidase expression by peripheral blood neutrophils. Although the two methods yielded comparable accuracy in diagnosing MDS, the area under the ROC curve, sensitivity and negative predictive value were found to be lower than previously published estimates.

Overall, the diagnostic performance attributes of peripheral blood neutrophil myeloperoxidase expression, as quantified by flow cytometric analysis, are supported by a retrospective case–control study and three prospective validation studies totalling 311 patients, of whom 119 (38%) received a reference diagnosis of MDS.^31^ However, the area under the ROC curve point estimates for BD Lyotube Stain 468 and its laboratory-developed liquid reagent counterpart were 0.82–0.83, which is lower than the 0.87–0.94 point estimates observed in the previous case–control derivation and prospective validation studies.^31^ Furthermore, using a prespecified threshold of 30%, the sensitivity point estimates for intra-individual RCV were 89% for BD Lyotube Stain 468 and 95% for the laboratory-developed liquid reagent-based assay compared with 100% in the previous prospective validation studies.^31^

The BD Lyotube Stain 468 yielded four false negative cases, whereas the laboratory-developed liquid reagent-based assay yielded two. In comparison, only one false negative case was reported in three previous studies. The underlying causes of these false negative results remain uncertain; however, they may be due to random inherent variability in intra-individual RCV. This hypothesis requires further investigation and a large multicentre prospective validation study is warranted to provide more precise estimates of diagnostic performance attributes.^32^

Cytomorphological evaluation of baseline or follow-up bone marrow aspirates provided an alternative diagnosis in four patients with an unconfirmed suspicion of MDS (6.3%). It is noteworthy that three of these patients were ultimately diagnosed with acute myeloid leukaemia, dendritic cell neoplasm or erythropenia, and had an intra-individual RCV exceeding 30% by either or both methods. Cytomorphological evaluation of the bone marrow aspirate was helpful in establishing an alternative diagnosis in a single true negative case (ie, an unconfirmed suspicion of MDS with an intra-individual RCV less than 30% with both methods). The only exception was a patient in whom bone marrow aplasia was diagnosed by repeated bone marrow aspiration after 156 days of follow-up. In light of these observations, the relevance of bone marrow aspiration or biopsy in patients with intra-individual RCV values below 30% is questionable.^12^

Intra-individual RCV of peripheral blood neutrophil myeloperoxidase expression may be a sufficiently sensitive test to safely rule out MDS on its own.^33^ While the routine use of BD Lyotube Stain 468 cannot be recommended at this time, it may be a suitable option for patients at increased risk of complications from bone marrow aspiration, those who refuse bone marrow aspiration and those in whom cytomorphological evaluation is uninterpretable.^22^ Prospective management studies or cluster randomised trials are needed to demonstrate the effectiveness of RCV in altering physician practice, without compromising patient safety.^34 35^

The present study has several notable strengths. First, cytomorphological evaluation of bone marrow for establishing the reference diagnosis was performed in duplicate by experienced haematopathologists who were blinded to the index test results.^22^ Second, the potential for spectrum bias was limited by enrolling unselected consecutive patients referred for suspected MDS.^36^ Third, a prespecified threshold for RCV (30.0%) was used to prevent reporting of optimistic diagnostic accuracy estimates.^36^ Fourth, RCV could be determined with both index tests for all patients, with no missing values, thereby supporting the validity of our diagnostic accuracy estimates.

The limitations of the present study deserve mention. First, the study sample may not encompass the full spectrum of MDS subtypes or prognosis. Therefore, it is possible that the BD Lyotube Stain 468 would have yielded different results in patients at higher risk. Reassuringly, we found a significant trend towards higher RCV values in patients with higher-risk MDS, as defined by the revised International Prognostic Scoring System.^28^

Second, bone marrow karyotype and molecular profiling were available in 73% (75/103) and 37% (38/103) of the study participants, respectively, because the reference diagnosis of MDS was based on cytomorphological evaluation of the bone marrow aspirate. Next-generation sequencing analysis was restricted to challenging suspicions of MDS where the detection of somatic mutations could facilitate diagnosis or prognostic assessment.

Third, the study was conducted in a single hospital laboratory at a university hospital, a fact which may limit the generalisability of the findings to other settings. Although the original laboratory-developed liquid reagent-based assay demonstrated satisfactory reproducibility,^10^ further investigation is required to ascertain the reproducibility of BD Lyotube Stain 468 across laboratories.

## Conclusion

This study suggests that a single-use flow cytometric lyophilised reagent tube performs as well as its laboratory-developed liquid reagent counterpart in quantifying myeloperoxidase expression by peripheral blood neutrophils. While BD Lyotube Stain 468 has the potential to save time and resources in busy flow cytometry laboratories, it requires further validation before its use can be recommended for ruling out MDS in routine practice.

## Supplementary material

10.1136/bmjopen-2024-095640 online supplemental file 1

## Data Availability

Data are available upon reasonable request.
