# Supplementary material for: Flow cytometric lyophilised reagent tube assay for peripheral blood neutrophil myeloperoxidase expression to rule out myelodysplastic neoplasms at a university hospital: a diagnostic accuracy study
Source: BMJ Open. 2025 Aug 22;15(8):e095640. doi: 10.1136/bmjopen-2024-095640 (PMC12374650; doi:10.1136/bmjopen-2024-095640)

Supplementary Table 1. Intra-assay precision estimates for robust coefficient of variation of neutrophil myeloperoxidase expression in peripheral blood quantified using BD Lyotube Stain 468™.[a]

	Assay					
Individual	1	2	3	Mean	(SD)	CV, %
1	30.0	30.3	29.8	30.0	(0.2)	0.8
2	28.7	29.9	28.9	29.2	(0.6)	2.2
3	35.1	34.1	33.9	34.4	(0.6)	1.9
4	29.9	30.6	29.7	30.1	(0.5)	1.6
5	28.4	29.1	29.4	29.0	(0.5)	1.8

Abbreviations: CV = coefficient of variation; SD = standard deviation.

[a] Blood samples were collected from five individuals. Each sample was assayed in triplicate in a single analytical run by the same operator. Values are robust coefficient of variation for peripheral blood neutrophil myeloperoxidase expression quantified using BD Lyotube stain 468™.

Supplementary Table 2. Inter-assay precision estimates for robust coefficient of variation of neutrophil myeloperoxidase expression in peripheral blood using BD Lyotube Stain 468™.[a]

Operator							
1	2	3	4	5	Mean	(SD)	CV, %
34.5	34.0	34.8	33.5	35.2	34.4	(0.7)	1.9

Abbreviations: CV = coefficient of variation; SD = standard deviation.

[a] A single blood sample from a single individual was assayed by five different operators in five independent analytical runs at the same day. Values are robust coefficient of variation for peripheral blood neutrophil myeloperoxidase expression quantified using BD Lyotube stain 468™

Supplementary Table 3. Specimen stability estimates for robust coefficient of variation of neutrophil myeloperoxidase expression in peripheral blood according to storage conditions and timing.[a]

		Storage of unprocessed specimen at 4°C			Storage of unprocessed specimen at room temperature		
Individual	Baseline	24 h	48 h	72 h	24 h	48 h	72 h
1	27.9	27.8	27.3	27.6	37.2	35.9	31.5
2	27.8	29.5	29.0	29.4	31.7	34.4	37.4
3	29.9	29.9	29.8	32.1	38.5	60.2	63.8
4	30.3	31.2	30.6	29.9	33.4	37.1	34.3
5	28.1	29.8	29.1	29.6	39.4	50.8	42.2
Mean (SD)	28.8 (1.2)	29.6 (1.2)	29.2 (1.2)	29.7 (1.6)	36.0 (3.3)	43.7 (11.3)	41.8 (12.9)
Mean difference[b]	…	0.8	0.4	0.9	7.2	14.9	13.0
(95% CI)	(…)	(-0.2 to 1.9)	(-0.6 to 1.3)	(-0.6 to 2.4)	(2.8 to 11.7)	(1.3 to 28.5)	(-2.4 to 28.5)

Abbreviations: CI = confidence interval; SD = standard deviation

[a] Blood samples from five individuals were assayed by the same operator at four different time points (i.e., baseline, 24 h, 48 h, and 72h) with storage of unprocessed specimens at 4°C and room temperature, respectively (See Methods). Values are robust coefficients of variation for peripheral blood neutrophil myeloperoxidase expression quantified using BD Lyotube stain 468™.

[b] Mean difference (95% confidence interval) in robust coefficient of variation for peripheral blood neutrophil myeloperoxidase expression value from baseline.

Supplementary Table 4. Clinical and laboratory features for patients with confirmed suspicions of myelodysplastic neoplasm (n = 37).

#	2022 WHO classification	BM blasts, %	Cytogenetic abnormalities	Somatic mutations	FCS[a]	R-IPSS	Baseline RCV, %[b] BD Lyotube stain 468™	Baseline RCV, %[b] Laboratory-developed assay
2	MDS-LB	2.7	7q deletion	*DNMT3A*, *U2AF1*, *STAG2*, *JAK2*	4	Low	41.7	46.2
3	MDS-LB	2.0	Complex karyotype[c]	Not performed	1	Low	34.4	36.8
4	MDS-IB1	7.7	Complex karyotype[d]	Not performed	3	Very high	31.1	32.9
10	MDS-*SF3B1*	1.2	Normal karyotype	*SF3B1*	…	Very low	35.3	36.1
11	MDS-LB	2.5	Normal karyotype	*TET2*, *RUNX1*	2	Low	37.7	38.3
12	MDS-LB	1.5	Normal karyotype	*SRSF2*, *IDH2*	2	Very low	47.4	49.2
13	MDS-LB	1.5	20q deletion	*U2AF1*	4	Very low	36.9	34.0
15	MDS-LB	2.0	Normal karyotype	*TET2*, *SRSF2*	3	Very low	33.8	37.6
28	MDS-LB	2.5	Normal karyotype	Not performed	3	Low	33.8	34.5
29	MDS-IB2	14	Partial Xq deletion	*TET2*, *SRSF2*	4	Very high	40.7	37.5
34	MDS-LB	4.2	Monosomy 7	*TET2*, *EZH2*	2	High	69.4	60.0
35	MDS-IB1	5.5	Normal karyotype	*TET2*, *ZRSR2*	1	Intermediate	34.6	32.8
38	MDS-LB-RS wild-type *SF3B1*	2.0	12q deletion	*ZRSR2*	0	Low	29.2[e]	30.1
39	MDS-bi*TP53*	3.7	Complex karyotype[f]	*TP53*	3	Very high	34.9	35.8

(Continued on next page)

Supplementary Table 4. (Continued)

#	2022 WHO classification	BM blasts, %	Cytogenetic abnormalities	Somatic mutations	FCS[a]	R-IPSS	Baseline RCV, %[b] BD Lyotube stain 468™	Baseline RCV, %[b] Laboratory-developed assay
42	MDS-IB2	14	Trisomy 8	*TET2*, *SRSF2*, *RUNX1*, *STAG2*	4	Very high	56.2	57.1
49	MDS-IB1[g]	6.0	Normal karyotype	*ASXL1*, *U2AF1*, *BCOR*, *BCORL1*	2	High	30.4	30.8
55	MDS-*SF3B1*	2.5	Normal karyotype	*SF3B1*, *DNMT3A*, *JAK2*	1	Intermediate	32.5	34.4
61	MDS-LB	0.7	Not performed	Not performed	4	Very low	39.0	39.0
63	MDS-LB	0.7	20q deletion	No mutation	3	Very low	33.6	32.4
70	MDS-LB	1.2	LOY	Not performed	3	Very low	36.6	33.4
71	MDS-*SF3B1*	1.7	Normal karyotype	*SF3B1*, *TET2*	1	Low	27.3[e]	29.0[h]
72	MDS-LB	1.5	Normal karyotype	*TET2*, *ASXL1*, *RUNX1*,	4	Very low	43.4	46.2
73	CMML1[i]	2.0	Normal karyotype	Not performed	1	Very low	30.8	31.2
80	MDS-IB2 (with Auer rods)	8.2	Normal karyotype	*TET2* (2), *ASXL1*, *BCOR*, *CEBPA*, *CSF3R*, *EZH2*, *JAK2*, *KRAS* (2), *NRAS* (3), *STAG2*	4	Intermediate	60.2	60.3
83	MDS-IB1	5.7	5q deletion	Not performed	2	Intermediate	38.4	37.5
85	MDS-LB	2.0	LOY	Not performed	2	Very low	29.2[e]	30.1

(Continued on next page)

Supplementary Table 4. (Continued)

#	2022 WHO classification	BM blasts, %	Cytogenetic abnormalities	Somatic mutations	FCS[a]	R-IPSS	Baseline RCV, %[b] BD Lyotube stain 468™	Laboratory-developed assay
89	MDS-IB2	15	Normal karyotype	*TET2*, *SRSF2*, *CBL*, *IDH1*, *MPL*, *STAG2*	3	Intermediate	34.9	33.5
96	CMML1[i]	3.7	Normal karyotype	*TET2* (2), *SRSF2*, *CUX1* (2)	3	Low	32.7	34.5
97	MDS-*SF3B1*	1.0	20q deletion	*SF3B1*	1	Very low	33.1	34.5
99	MDS-LB	3.2	Complex karyotype[j]	Not performed	0	Very high	30.3	31.9
101	MDS-IB2	11	Complex karyotype[k]	Not performed	…	Very high	32.2	31.8
104	CMML1[i]	0.7	Normal karyotype	*ASXL1*, *CBL*	3	Very low	31.5	30.8
106	MDS-IB1[l]	7.0	Complex karyotype[m]	*TP53*, *PPM1D*	1	High	25.0[e]	25.9[h]
109	CMML1[i]	2.0	Normal karyotype	*TET2* (2), *ZRSR2*	2	Very low	54.5	50.0
114	MDS-LB	4.2	Complex karyotype[n]	*TP53*	3	High	57.8	53.5
119	MDS-LB	3.2	Normal karyotype	*TET2*, *RUNX1* (2), *BCOR* (2), *EZH2*, *PTPN11*	2	Intermediate	40.0	39.1
120	MDS-LB	4.2	Trisomy 15, LOY	Not performed	2	Low	35.3	30.9

Abbreviations: BM, bone marrow; CMML1, chronic myelomonocytic leukaemia 1; FCS, flow cytometric score; R-IPSS, revised-international prognostic scoring system; LOY, loss of Y chromosome; MDS-bi*TP53*, myelodysplasic neoplasm with biallelic *TP53* inactivation; MDS-IB1, myelodysplasic neoplasm with increased blasts 1; MDS-IB2, myelodysplasic

neoplasm with increased blasts 2; MDS-LB, myelodysplasic neoplasm with low blasts; MDS-LB-RS, myelodysplasic neoplasm with low blasts and ring sideroblasts; MDS-*SF3B1,* myelodysplasic neoplasm with low blasts and *SF3B1* mutation; RCV, robust coefficient of variation; WHO, World Health Organization.

[a] Bone marrow flow cytometric score was computed as the number of parameters with values outside of the reference ranges among myeloblast-related cluster size in all nucleated cells (%), B-progenitor-related cluster size in all CD34+ cells (%), lymphocytes to myeloblast CD45 ratio, and granulocyte to lymphocyte SSC ratio for an individual.[25]

[b] Robust coefficient of variation for peripheral blood neutrophil myeloperoxidase expression quantified using the single-use flow cytometry tube of lyophilized reagents (BD Lyotube stain 468™) and its laboratory-developed liquid reagent counterpart (See Methods).

[c] Complex karyotype for patient #3 was: 46,XX,del(5)(q13q34)[4]/ 46,sl,del(11)(q21q25)[7]/ 46,sdl1,add(17)(q22)[6]/ 46,XX[3]

[d] Complex karyotype for patient #4 was: 46,XY,add(3)(q11),add(4)(q25),-5,?add(6)(p21),add(7)(q22),+8,-14,?add(17)(p12),+mar,inc[cp7]/ 46,XY[2]

[e] Using a 30% prespecified threshold for RCV, there were four false negative cases using BD Lyotube Stain 468 ™. The median time from peripheral blood collection to flow cytometric analysis was 7h24 (range, 3h02 to 10h15) for these four patients.

[f] Complex karyotype for patient #39 was: 43-44,XY,-3[9],der(4)add(4)(p12)del(4)(q2?1q2?5)[9],del(5)(q14q33)[11],del(7)(q32q36)[10],del(8)(q21q24) [8],add(12)(p12)[2] or der(12)t(3;12)(q12;p12)[4],del(15)(q24q26)[9],add(17)(q25)[5],-20[11][cp11]/46,XY[1]

[g] Although this patient was initially categorized as idiopathic cytopenia of undetermined significance (ICUS) at baseline, the reference diagnosis was MDS-IB1 after cytomorphologic evaluation of bone marrow aspirate repeated by day 304 of follow-up.

[h] Using a 30% prespecified threshold for RCV, there were two false negative cases using the laboratory-developed liquid reagent assay. Time to peripheral blood flow cytometric analysis was 10h15 for these two patients.

[i] MO1 fraction over 94% of total monocytes

[j] Complex karyotype for patient #99 was: 46,XY,+8,-11,-22,+mar[3]/46,XY[4]

[k] Complex karyotype for patient #101 was: 50,XX,+X,+1,del(5)(q14q34),+11,+22[10]

[l] Although this patient was initially categorized as clonal cytopenia of undetermined significance (CCUS) at baseline, the reference diagnosis was MDS-IB1 after cytomorphologic evaluation of bone marrow aspirate repeated by day 147 of follow-up.

[m] Complex karyotype for patient #106 was: 44,XX,-5,-11,-17,-20,+mars[6]/46,XX[14]

[n] Complex karyotype for patient #114 was: 45-47,XX,-1[3],-3[3],add(3)(q26)[4],del(5)(q21q34)[8],del(6)(q12q27)[3],-7[3],add(11)(p15)[2],add(11)(q23) [4],-12[3],add(12)(p12)[4],+mar[6][cp8]/46,XX[2].ish t(3;3)(3'MECOM+,3'MECOM+,5'MECOM+;5'MECOM+)

Supplementary Table 5. Clinical and laboratory features for patients with clonal or idiopathic cytopenia of undetermined significance.

#	Reference diagnosis	BM blasts, %	Cytogenetic abnormalities	Somatic mutations	Baseline RCV, %[a] BD Lyotube stain 468™	Laboratory-developed assay	Follow-up BM aspirate	Time, *d*
19	ICUS	0.5	Normal karyotype	Not performed	27.9	27.2	Not performed	…
23	CCUS	1.7	11q deletion	Not performed	31.9	35.4	Uninterpretable	104
36	ICUS	3.0	Normal karyotype	No mutation	25.6	25.9	ICUS	54
40	ICUS	1.2	Normal karyotype	Not performed	26.5	27.8	Not performed	…
81	CCUS	1.7	Normal karyotype	*DNMT3A*, *TP53*	31.0	31.1	Not performed	…
87	CCUS	1.0	Normal karyotype	*DNMT3A*	25.2	26.6	Not performed	…
111	CCUS	1.2	Normal karyotype	*DNMT3A*, *TET2*	28.1	26.9	CCUS	16
113	CCUS	1.7	Normal karyotype	*TET2*	40.9	33.4	CCUS	11

Abbreviations: BM, bone marrow; CCUS, clonal cytopenia of undetermined significance; ICUS, idiopathic cytopenia of undetermined significance; RCV, robust coefficient of variation.

[a] Robust coefficient of variation for peripheral blood neutrophil myeloperoxidase expression quantified using the single-use flow cytometry tube of lyophilized reagents (BD Lyotube stain 468™) and its laboratory-developed liquid reagent counterpart (See Methods).

Supplementary Table 6. Clinical and laboratory features for patients with uninterpretable baseline bone marrow aspirate (n = 3).

#	Baseline BM aspirate	Baseline RCV, %[a] BD Lyotube stain 468™	Baseline RCV, %[a] Laboratory-developed assay	Follow-up BM aspirate	Time, *d*
33	Uninterpretable	27.9	28.3	Not performed	…
82	Uninterpretable	25.9	27.6	Uninterpretable	62
102	Uninterpretable	32.2	32.8	Not performed	…

Abbreviations: BM, bone marrow; RCV, robust coefficient of variation.

[a] Robust coefficient of variation for peripheral blood neutrophil myeloperoxidase expression quantified using the single-use flow cytometry tube of lyophilized reagents (BD Lyotube stain 468™) and its laboratory-developed liquid reagent counterpart (See Methods).

Supplementary Table 7. Clinical and laboratory features for patients with alternate diagnosis other than myelodysplastic neoplasm established by bone marrow aspirate ($n = 4$).

#	BM blasts, %	Alternate diagnosis	Baseline RCV, %[a] BD Lyotube stain 468™	Baseline RCV, %[a] Laboratory-developed assay
103	22.5	Acute myeloid leukemia	35.4	31.6
108	39.2	Dendritic cell neoplasm	35.1	43.4
112	0.7	Bone marrow aplasia on bone marrow aspirate repeated by 156 days of follow-up	29.2	28.6
118	1.7	Erythropenia on bone marrow aspirate repeated by 300 days of follow-up	29.5	32.3

Abbreviations: BM, bone marrow; RCV, robust coefficient of variation.

[a] Robust coefficient of variation for peripheral blood neutrophil myeloperoxidase expression quantified using the single-use flow cytometry tube of lyophilized reagents (BD Lyotube stain 468™) and its laboratory-developed liquid reagent counterpart (See Methods).

Supplementary Table 8. Comparison of baseline clinical and laboratory features for participants with confirmed and unconfirmed suspicions of myelodysplastic neoplasm (*n* = 100).[a]

Characteristics[b]	Suspicion of MDS: Unconfirmed (*n* = 63)		Suspicion of MDS: Confirmed (*n* = 37)		*P*
Female gender, *n (%)*	25	(40)	15	(41)	.93
Age, median (IQR), *y*	74	(67–82)	76	(68–81)	.77
Smoking status, *n (%)*					.99
Never smoked	37	(59)	22	(59)	
Former smoker	21	(33)	13	(35)	
Current smoker	5	(7.9)	2	(5.4)	
Alcohol use disorders, *n (%)*	9	(14)	5	(14)	.91
Occupational exposure to chemicals, *n (%)*	1	(1.6)	2	(5.4)	.55
History of hematological disease, *n (%)*[c]					
Lymphoproliferative syndrome	1	(1.6)	1	(2.7)	.99
Multiple myeloma	1	(1.6)	0	(…)	.99
Other	0	(…)	2	(5.4)	.14
History of immunosuppressive or biological therapy, *n (%)*	8	(13)	0	(…)	.03
Hemoglobin, median (IQR), *g/dL*	10.2	(9.2–12.5)	10.6	(9.1–11.1)	.73
Platelet, median (IQR), ×*10^9/L*	153	(89–219)	108	(73–190)	.16
WBC, median (IQR), ×*10^9/L*	5.2	(3.5–7.1)	4.4	(3.2–5.8)	.16
ANC, median (IQR), ×*10^9/L*	3.5	(2.1–4.7)	2.4	(1.8–3.3)	.04
Lymphocytes, median (IQR), ×*10^9/L*	1.1	(0.8–1.7)	1.3	(0.9–1.8)	.46
Monocytes, median (IQR), ×*10^9/L*	0.4	(0.3–0.6)	0.3	(0.2–0.8)	.46
Reticulocytes, median (IQR), ×*10^9/L*	74	(48–96)	72	(55–100)	.95
Creatinine, median (IQR), *µmol/L*	77	(58–107)	70	(58–98)	.47
Blood urea nitrogen, median (IQR), *µmol/L*	6.9	(5.2–11)	6.2	(4.8–10)	.31
Bilirubin, median (IQR), *µmol/L*	11	(7–17)	11	(8–17)	.58

(Continued on next page)

Supplementary Table 8. **(Continued)**

Characteristics[b]	Suspicion of MDS				*P*
	Unconfirmed (*n* = 63)		Confirmed (*n* = 37)		
AST, median (IQR), *IU/L*	25	(20–36)	24	(16–34)	.53
ALT, median (IQR), *IU/L*	22	(16–28)	22	(15–33)	.43
Gamma-glutamyltransferase, median (IQR), *IU/L*	50	(24–108)	35	(20–63)	.07
Alkaline phosphatase, median (IQR), *IU/L*	82	(65–125)	70	(50–89)	.01
Serum folic acid, median (IQR), *ng/mL*	8.8	(6.3–16)	9.6	(6.8–13)	.84
Serum B12 vitamin, median (IQR), *pg/mL*	433	(350–576)	592	(391–841)	.03
TSH, median (IQR), *mIU/L*	1.61	(0.98–2.25)	1.74	(1.29–2.63)	.38
Haptoglobin, median (IQR), *g/L*	1.27	(0.71–1.92)	0.89	(0.54–1.38)	.08
Ferritin, median (IQR), *ng/mL*	199	(84–478)	312	(129–516)	.23
C-reactive protein, median (IQR), *mg/L*	0	(0–22)	0	(0–10)	.15

Abbreviations: ALT, alanine aminotransferase; AST, aspartate aminotransferase; ANC, absolute neutrophil count; IQR, interquartile range (25–75th percentiles); MDS, myelodysplastic neoplasm; TSH, thyroid-stimulating hormone; WBC, white blood cell.

[a] Bone marrow cytomorphology was uninterpretable for three patients (See Figure 1).

[b] Values were missing for blood urea nitrogen (*n* = 2), bilirubin (*n* = 2), alkaline phosphatase (*n* = 1), gamma-glutamyltransferase (*n* = 1), serum folic acid (*n* = 2), TSH (*n* = 1), and C-reactive protein (*n*=1) concentration.

[c] No patient reported history of acute leukemia, myeloproliferative neoplasms, idiopathic thrombocytopenic purpura, familial hematological malignancies, antineoplastic chemotherapy, or radiation therapy. Other hematological diseases included minor thalassemia (*n* = 1) and hemochromatosis (*n* = 1).

Supplementary Table 9. Agreement of binary intra-individual robust coefficient of variation for peripheral blood neutrophil myeloperoxidase expression between a single-use flow cytometric lyophilized reagent tube (BD Lyotube stain 468™) and its laboratory-developed liquid reagent counterpart (Kappa coefficient, 0.72, 95% confidence interval, 0.59 to 0.86).

Laboratory-developed liquid reagent test	BD Lyotube stain 468™ RCV < 30%	BD Lyotube stain 468™ RCV ≥ 30%	Total
RCV < 30%	35	3	38
RCV ≥ 30%	11	54	65
Total	46	57	103

Abbreviations: RCV = robust coefficient of variation.

Supplementary Figure 1. Agreement in continuous intra-individual robust coefficient of variation for peripheral blood neutrophil myeloperoxidase expression between a single-use flow cytometric lyophilized reagent tube (BD Lyotube stain 468™) and its laboratory-developed liquid reagent counterpart (n = 103).

Abbreviations: RCV, robust coefficient of variation

* Mean difference of laboratory-developed liquid reagent-based assay RCV minus BD Lyotube Stain 468™ RCV

† β regression coefficient for slope estimate was -0.07 (95% confidence interval, -0.14 to -0.01, *P* = .03).

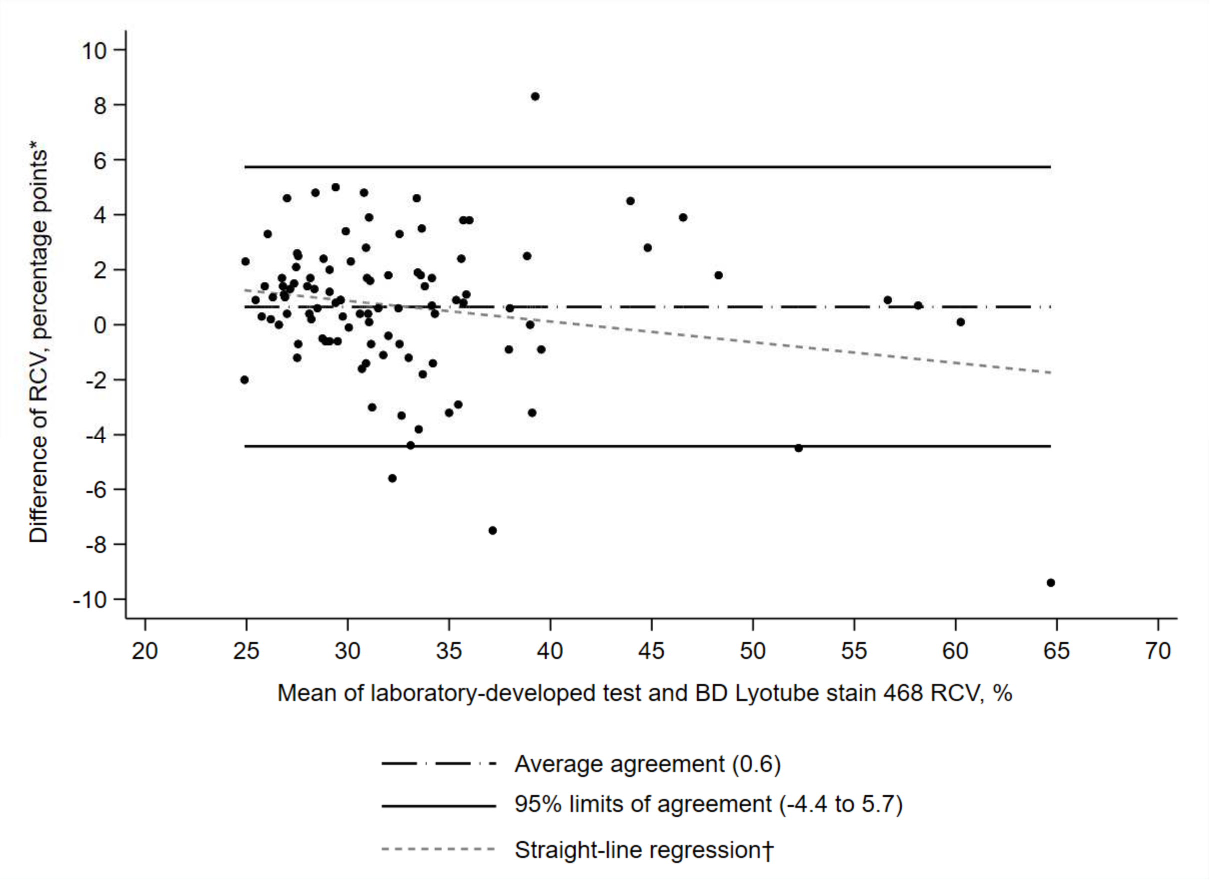

Supplementary Figure 2. Trends in continuous intra-individual robust coefficient of variation for peripheral blood neutrophil myeloperoxidase expression quantified using a single-use flow cytometric lyophilized reagent tube (BD Lyotube stain 468™) and its laboratory-developed liquid reagent counterpart according to the revised International Prognostic Scoring System (n = 100).*

Abbreviations: MDS, myelodysplastic neoplasm.

* The analytical sample consisted of 37 confirmed and 63 unconfirmed suspicions of myelodysplastic neoplasm, after excluding three patients with uninterpretable bone marrow cytomorphology. The low risk class included very low, low, and intermediate categories while the high risk class included high and very high categories of the revised International Prognostic Scoring System.

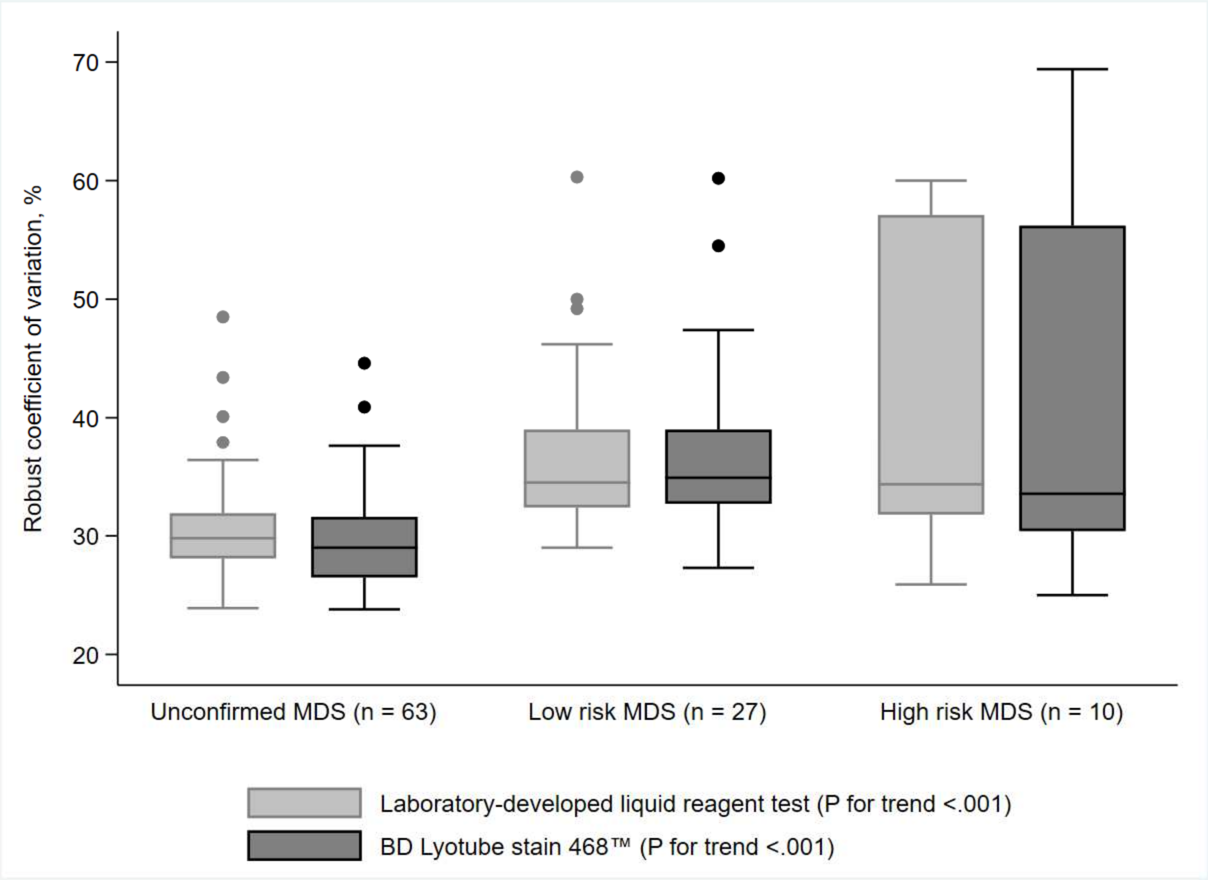

Supplement: online supplemental file 1 [file bmjopen-15-8-s001.pdf]
