# Supplementary material for: Flow cytometric lyophilised reagent tube assay for peripheral blood neutrophil myeloperoxidase expression to rule out myelodysplastic neoplasms at a university hospital: a diagnostic accuracy study
Source: BMJ Open. 2025 Aug 22;15(8):e095640. doi: 10.1136/bmjopen-2024-095640 (PMC12374650; doi:10.1136/bmjopen-2024-095640)
Supplement: Supplementary file 2 [file bmjopen-2024-095640.reviewer_comments.pdf]

PEER REVIEW HISTORY

BMJ Open publishes all reviews undertaken for accepted manuscripts. Reviewers are asked to complete a checklist review form and are provided with free text boxes to elaborate on their assessment. These free text comments are reproduced below.

ARTICLE DETAILS

Title (Provisional)

Flow cytometric lyophilised reagent tube assay for peripheral blood neutrophil myeloperoxidase expression to rule out myelodysplastic neoplasms at a university hospital. A diagnostic accuracy study.

Authors

Planta, Charlotte; Scheffen, Laura; Jacob, Marie-Christine; Szymanski, Gautier; Chevalier, Simon; Tondeur, Sylvie; Bulabois, Bénédicte; Meunier, Mathieu; Lefebvre, Christine; Gonnet, Nicolas; Molina, Lysiane; Paradis, Claire; Chiriac, Radu; Labarère, José; Garban, Frédéric; Seigneurin, Arnaud; Merle, Raymond; Park, Sophie; Raskovalova, Tatiana

---

VERSION 1 - REVIEW

**Reviewer**	**1**
**Name**	**Podvin, Benjamin**
**Affiliation**	**Lille University Hospital**
**Date**	**31-Mar-2025**
**COI**	**None**

The manuscript is clear and well written. The authors present the performance of a lyophylated tube for assessing phenotypic expression of MPO by flow cytometry. The data are well presented, and the tube's performance is clearly demonstrated. The study has certain limitations: monocentric, complementary examinations not always available, however the results are interesting and promising for simplifying laboratory workflow for MPO evaluation by flow cytometry.

**Reviewer**	**2**
**Name**	**Psarra, Katherina**
**Affiliation**	**Evangelismos Athens General Hospital**
**Date**	**21-Apr-2025**
**COI**	**None**

This is an interesting study concerning the comparison of wet and dried reagents combinations for a very practical approach for the diagnosis of MDS in the peripheral blood of patients referred as possible MDS. It is very important to help in the diagnosis without the need of bone marrow samples and a dry tube will contribute to the reproducible results by many centres. The methods and results concerning flow cytometry seem fine to me. I am not an expert in the statistical approach. It looks fine to me but perhaps a statistician might be more suitable for this estimation.

Reviewer 3

Name Sroka, Christopher

Affiliation New Mexico State University, Department of Economics, Applied Statistics and International Business

Date 04-Jun-2025

COI None

My review focused solely on the statistical analysis. I found the statistical methods used in the paper to be sound and appropriate for the research question. Results were clearly presented. The conclusions were justified based on the statistical results.

I have a few very minor suggestions that would improve the clarity of the manuscript. First, it is not clear what data were missing and why, and which analyses were affected by missing data and which ones were not. It was only looking at the Supplementary Tables that it was clear that very few observations were missing, and that they were in covariates in the logistic regression. While the authors did mention that MDS diagnosis and RCV were never missing, this should be emphasized more because these are the most important variables. Given this, the missing data is almost a non-issue. The paper can be strengthened by noting how the key results are not dependent on the missing data or the method for addressing the missing values.

It was not clear in the text how the 3 cases with indeterminate MDS were handled in the analysis. Again, it was only when looking at the results that this became clear. I applaud the authors for having a footnote in Figure 2 clearly stating that those cases were discarded (all researchers should follow this example of providing clear footnotes to tables and figures so that they can stand alone apart from the text). I could not find a similar statement in the text, and it would be helpful to include one.

I suggest assigning a different name to the "Reference" line in Figure 2 to avoid confusion with the reference test conducted in the study. At first glance, it seems the three lines represent the three different tests (two index tests and the reference test). The 45 degree line in the ROC plot represents the worse case scenario, one where a person randomly assigns a diagnosis. This is not the same as the reference test which (hopefully) has better

sensitivity and specificity than a random coin toss. Some suggested labels are "Minimum achievable sensitivity and specificity" or "45 degree line".

VERSION 1 - AUTHOR RESPONSE

Reviewer 1

The manuscript is clear and well written. The authors present the performance of a lyophylated tube for assessing phenotypic expression of MPO by flow cytometry. The data are well presented, and the tube's performance is clearly demonstrated. The study has certain limitations: monocentric, complementary examinations not always available, however the results are interesting and promising for simplifying laboratory workflow for MPO evaluation by flow cytometry.

We are grateful to the Reviewer for his kind appreciation of our manuscript.

Reviewer 2

This is an interesting study concerning the comparison of wet and dried reagents combinations for a very practical approach for the diagnosis of MDS in the peripheral blood of patients referred as possible MDS. It is very important to help in the diagnosis without the need of bone marrow samples and a dry tube will contribute to the reproducible results by many centres. The methods and results concerning flow cytometry seem fine to me. I am not an expert in the statistical approach. It looks fine to me but perhaps a statistician might be more suitable for this estimation.

We are grateful to the Reviewer for her kind appreciation of our manuscript. We would like to inform this Reviewer that one of the authors (JL) is a senior statistician.

Reviewer 3

My review focused solely on the statistical analysis. I found the statistical methods used

in the paper to be sound and appropriate for the research question. Results were clearly presented. The conclusions were justified based on the statistical results.

We would like to express our gratitude to this Reviewer for his constructive feedback on our manuscript.

1. ***I have a few very minor suggestions that would improve the clarity of the manuscript. First, it is not clear what data were missing and why, and which analyses were affected by missing data and which ones were not. It was only looking at the Supplementary Tables that it was clear that very few observations were missing, and that they were in covariates in the logistic regression. While the authors did mention that MDS diagnosis and RCV were never missing, this should be emphasized more because these are the most important variables. Given this, the missing data is almost a non-issue. The paper can be strengthened by noting how the key results are not dependent on the missing data or the method for addressing the missing values.***

As suggested by this Reviewer, we emphasized the fact that RCV obtained with both methods could be determined for all patients, with no missing values, thereby supporting the validity of our estimates. Furthermore, we have made the low prevalence of missing values for covariates (<2%) more explicit, resulting in consistent odds ratio estimates, with or without multiple imputations of missing values. Supplementary Table 4 has also been deleted due to its redundancy with Table 1.

The following section details the alterations made to the manuscript in response to the aforementioned comments:

"Given that the prevalence of missing values was less than 2% for covariates (Table 1), the odds ratio estimates were consistent with and without multiple imputation of missing values."

Results section, page 17

“Fourth, RCV could be determined with both index tests for all patients, with no missing values, thereby supporting the validity of our diagnostic accuracy estimates.” Discussion, pages 19–20

2. ***It was not clear in the text how the 3 cases with indeterminate MDS were handled in the analysis. Again, it was only when looking at the results that this became clear. I applaud the authors for having a footnote in Figure 2 clearly stating that those cases were discarded (all researchers should follow this example of providing clear footnotes to tables and figures so that they can stand alone apart from the text). I could not find a similar statement in the text, and it would be helpful to include one.***

To address this comment from the Reviewer, we have incorporated the following sentence in the Results section:

“The analytical sample for diagnostic accuracy of RCV consisted of 37 confirmed and 63 unconfirmed suspicions of MDS, after excluding three patients with uninterpretable bone marrow cytomorphology (Figure 1).” Results section, pages 16–17

3. ***I suggest assigning a different name to the "Reference" line in Figure 2 to avoid*** **confusion with the reference test conducted in the study. At first glance, it seems the three lines represent the three different tests (two index tests and the reference test). The 45 degree line in the ROC plot represents the worse case scenario, one where a** ***person randomly assigns a diagnosis. This is not the same as the reference test which (hopefully) has better sensitivity and specificity than a random coin toss. Some suggested labels are "Minimum achievable sensitivity and specificity" or "45 degree line".***

In response to the Reviewer suggestion, we have renamed the random classifier line as "45-degree line" in Figure 2.
